# Loschmidt echo and time reversal in complex systems

**DOI:** 10.1098/rsta.2015.0383

**Published:** 2016-06-13

**Authors:** Arseni Goussev, Rodolfo A. Jalabert, Horacio M. Pastawski, Diego A. Wisniacki

**Affiliations:** 1 Department of Mathematics and Information Sciences, Northumbria University, Newcastle Upon Tyne NE1 8ST, UK; 2 Institut de Physique et Chimie des Matériaux de Strasbourg, Université de Strasbourg, CNRS UMR 7504, Strasbourg 67034, France; 3 Instituto de Física Enrique Gaviola (CONICET-UNC) and Facultad de Matemática, Astronomía y Física, Universidad Nacional de Córdoba, Córdoba 5000, Argentina; 4 Departamento de Física and IFIBA, FCEyN, UBA Ciudad Universitaria, Pabellón 1, Ciudad Universitaria, Buenos Aires 1428, Argentina

**Keywords:** reversibility, chaos, quantum, classical, semiclassical

## Abstract

Echoes are ubiquitous phenomena in several branches of physics, ranging from acoustics, optics, condensed matter and cold atoms to geophysics. They are at the base of a number of very useful experimental techniques, such as nuclear magnetic resonance, photon echo and time-reversal mirrors. Particularly interesting physical effects are obtained when the echo studies are performed on complex systems, either classically chaotic, disordered or many-body. Consequently, the term Loschmidt echo has been coined to designate and quantify the revival occurring when an imperfect time-reversal procedure is applied to a complex quantum system, or equivalently to characterize the stability of quantum evolution in the presence of perturbations. Here, we present the articles which discuss the work that has shaped the field in the past few years.

## 1. Introduction

The concept of time reversal has captured the imagination of physicists for centuries, leading to numerous vivid discussions. An emblematic example of these was the controversy around the second law of thermodynamics between Joseph Loschmidt and Ludwig Boltzmann. Loschmidt argued that, due to the time-reversal invariance of classical mechanics, evolution in which the entropy can decrease must exist. These states could be reached by reversing the velocities of all of the molecules of the system. Boltzmann’s response was that such a time-reversal experiment would be impossible for thermodynamic systems. Notwithstanding, today’s technological advances have made it possible to carry out time-reversal protocols in systems with few degrees of freedom that, although imperfect, provide a fascinating approach for studying the limitations of reversibility. Physical realizations that for a long time could only be discussed as ‘Gedankenexperiment’ are now readily implemented in the laboratory.

Many approaches to time reversal have the set-up of an echo experiment, in which an initial state is propagated for a given time and then reversed. The comparison of the resulting state and the initial one constitutes a measure of the irreversibility suffered by the system during its evolution and generated by differences between the forward and backward dynamics within the propagation medium. In quantum mechanics, this concept can be quantified through the measure of the fidelity, defined as
1.1
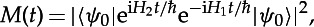
where |*ψ*_0_〉 is the state of the system at time 0, *H*_1_ is the Hamiltonian governing the forward evolution, *H*_2_ is the Hamiltonian governing the backward evolution and *t* is the instant at which the reversal takes place. This measure was initially proposed by Peres in 1984 [1] in his attempt to understand the origin of irreversibility in quantum mechanics. He focused on the differences in the long-time behaviour of the fidelity in a single-particle system stemming from the nature of the underlying classical dynamics, be it regular or chaotic. Subsequent work in the field emphasized the importance of the complexity of the medium in which the propagation takes place, and the term ‘Loschmidt echo’ was coined in order to describe the fidelity resulting from complex Hamiltonians. Such complexity can arise, for instance, from many-body aspects of the evolution of a spin system [2,3], or from the chaotic nature of the underlying classical dynamics.

In 2001, Jalabert and Pastawski [4] found that, for classically chaotic systems, the decay of the Loschmidt echo is typically determined solely by the properties of the unperturbed Hamiltonian, and can thus be independent of the perturbation strength beyond some threshold. Following a short-time parabolic decay, the Loschmidt echo exhibits an exponential decay, where two different regimes can be observed depending on the perturbation strength. For weak perturbations, the decay rate of the Loschmidt echo depends on the perturbation strength, while for stronger perturbations there is a transition to a perturbation-independent regime characterized by a decay rate equal to the average Lyapunov exponent of the underlying classical system.

The perturbation-independent regime (commonly referred to as the Lyapunov regime) was predicted using a semiclassical theory of the Loschmidt echo that relied on an ensemble-averaging over localized initial states and on the validity of the well-known ‘diagonal approximation’. The original studies pointed to the importance of classically adapted initial states for observing the Lyapunov regime [4–6]. Subsequent analytical and numerical approaches considered this crucial aspect, as well as the range of validity of the semiclassical approach. This endeavour generated a substantial body of work on different aspects of the physics and the phenomenology of the Loschmidt echo, which have been reviewed in the last decade [7–9].

Technical aspects of the Loschmidt echo and the rich variety of regimes for fidelity decay have been described in [7], which addresses the role of the initial state (localized wave-packets versus random states) and the type of dynamics (integrable versus chaotic dynamics). In addition, the random matrix description of the perturbation-dependent regime and the concept of ‘scattering fidelity’ (related to the standard fidelity and obtainable from scattering data) were discussed, together with the application of fidelity to quantum information as well as to experiments with microwave cavities and elasto-dynamic systems.

As discussed in [8], the Loschmidt echo provides a useful concept to study the quantum-to-classical transition for systems with few degrees of freedom. Connections with decoherence, entanglement and irreversibility could then be established in certain model systems. In addition, this review highlighted the importance of mesoscopic fluctuations of the Loschmidt echo, as well as the pertinence of considering other kinds of related echoes in order to describe the available experimental results.

Goussev *et al*. [9] presented the historical developments leading to the concept of the Loschmidt echo, its broad interest for seemingly unrelated domains, the various numerical tests of the semiclassical predictions and an overview of the relevant experiments. The main physical principles involved in the Loschmidt echo and the various decay regimes were analysed, stressing important issues such as the insight provided by a phase-space representation and the different behaviour obtained under global or local perturbations.

The above-mentioned connection of the quantum Loschmidt echo with classical chaos contributed to a sustained interest within different research domains, such as quantum chaos, solid-state physics, acoustics and cold atom physics. One common feature in these studies has been the effect of the complexity of the system, and/or the propagation medium, on the reconstruction quality of quantum states.

Initially, the studies were mainly focused on one-body aspects of the problem and the complexity stemmed from the chaotic character of the underlying classical dynamics or from the effect of disorder in the propagation medium. The focus of later work has shifted towards systems where the complexity arises from a many-body character and/or the interactions with a non-trivial environment.

The investigated many-body aspects include, for instance, the use of the Loschmidt echo as a sensitive probe of a quantum phase transition [10], as well as the connection between the Loschmidt echo and the statistics of the work done by a quantum critical system when a control parameter is quenched [11]. Moreover, the connections of the Loschmidt echo with decoherence phenomena and with the time reversal of waves have been fully explored and clarified.

This theme issue aims to update the existing body of knowledge with the recent developments in the domain. It is meant to provide a useful reference for researchers working in the field, as well as an optimal entry point for those starting to work on it. Each of the contributions tackles a particularly important aspect that helped in advancing our understanding of echoes in complex systems.

The method of semiclassical (short-wavelength) approximations has long been one of the most fruitful tools for exploring the physics of the Loschmidt echo and for understanding many important aspects of its behaviour. The main idea of the method is to describe quantum-mechanical processes by only using the information about the underlying classical system [12]. Tomsovic’s article [13] highlights and analyses a paradox pertinent to the semiclassical description: how can reversible and stable quantum motion be represented by classical dynamics that is essentially irreversible and unpredictable?

In practice, the semiclassical approach often suffers from well-recognized difficulties, such as the root search problem and the exponential growth of the number of classical orbits needed for a sufficiently accurate semiclassical expansion. A semiclassical formulation of the Loschmidt echo that allows one to overcome these difficulties is the so-called dephasing representation. The contribution of Vaníček & Cohen [14] provides a rigorous derivation of the dephasing representation based on the path integral formalism. The authors also construct higher order approximations of the quantum fidelity that resolve several shortcomings of the standard dephasing representation.

The contribution of García-Mata *et al.* [15] revisits the important problem of the dependence of the Lyapunov regime on the initial state over which the averaging is performed. Based on previous work addressing quantum maps [16], the authors introduce a modified measure that is independent of the initial state, thus allowing them to efficiently capture the Lyapunov regime. Such a measure, which is related to the Fourier transform of the work probability distribution after a quench [11], is essentially an average over initial states according to the Haar measure. These concepts are implemented in a model system: the paradigmatic Bunimovich stadium billiard.

The contribution of Gorin *et al.* [17] addresses a system which is coupled to a composite environment modelled by a random matrix model. The authors show that the coherences in the central system are given by fidelity amplitudes of a certain perturbed echo dynamics in the composite environment.

Wimberger’s article [18] reviews some of the recent advances in using echoes as a measure of complexity of the quantum motion in single- and many-body systems. Here, special attention is devoted to the problem of detecting avoided energy-level crossings in complex quantum systems and to the use of the Loschmidt echo in characterizing the motion of kicked ultracold atoms.

Žunkovič *et al*. [19] follows the strategy initiated by Quan *et al.* [10] of using a particular form of the Loschmidt echo, namely the survival or return probability of an unperturbed state after a sudden perturbation turn-on (quench), as a tool to test quantum phase transitions. By considering the echo amplitude in an infinite-range XY model and focusing on the first echo minimum, i.e. the survival collapse [20], they concluded that dynamical transitions are not necessarily connected to equilibrium quantum phase transitions.

The contribution of Engl *et al.* [21] extends the semiclassical approach to echoes used in the one-particle case into physical systems consisting of many interacting bosons. This new formalism is used to study the effect a coherent backscattering due to quantum interference in Fock space. The analogies and differences between the single- and the many-body semiclassical approximations are highlighted.

Inspired by the pioneering experimental echo study of the spin diffusion in nuclear magnetic resonance (NMR) [3], Zangara *et al.* [22] define the Loschmidt echo in terms of local and global observables for systems of different sizes. In particular, they verify that the corresponding time scales satisfy an extensiveness relation, which is consistent with the experimentally reached conclusion that a local irreversibility time scale could emerge as perturbation-independent quantity. They also suggest a connection of the Loschmidt echo with the scrambling of quantum information, occurring when a localized perturbation is spread across a quantum many-body system’s degrees of freedom and making the initial information inaccessible to local measurements. While, in the present case, the scrambling of quantum information is related to a divergent multi-spin correlation function, it is interesting to notice that this concept has been linked with the black hole information paradox, which is a problem of growing interest [23,24].

After a brief but comprehensive review of the whole variety of Loschmidt echo experiments in NMR, Sánchez *et al.* [25] focus on the multi-spin dynamics of two systems with completely different topologies. They show that the Loschmidt echo is an excellent quantifier of decoherence that, for example, allows one to enhance the assessment of how multi-spin entanglement, known as multiple quantum coherences, is created. Additionally, their results exemplify that the Loschmidt echo depends on the network of interactions which, in turn, reflect the underlying molecular structure.

The physics of echoes and time reversal has long been the focus of experimental studies with acoustic, elastic, water, electromagnetic and quantum-matter waves. Among them, there are the experiments with microwave billiards, which played a very important role in the development of the field of quantum chaos and provided compelling evidence for many theoretically predicted decay regimes of the Loschmidt echo. Kuhl’s article [26] reviews the concept and applications of the scattering fidelity. The scattering fidelity is often used in microwave experiments as an echo measure, since, under the conditions of an underlying chaotic dynamics and a weak coupling of the measuring antenna, it approaches the ordinary fidelity. The versatility of the microwave cavities allowed for an investigation of the response of complex systems (chaotic, disordered or localized) to perturbations of various types and strengths. Global and local perturbations induced by a moving piston on a boundary are considered in this article, as well as the perturbation induced by the coupling to the environment.

In addition to the above-described echo set-ups, the temporal inversion has also been implemented with acoustic, electromagnetic and water wave-fields. The concept of ‘time-reversal mirrors’ has been used to describe the reconstruction of an initial signal that is recorded, digitized, stored and time-reverse broadcasted by an antenna array. Time-reversal mirrors, based on the manipulation of the wave-field along a spatial boundary sampled by a finite number of antennas, are described by Fink’s article [27], together with the newly developed ‘instantaneous time mirrors’, where the waves are manipulated from a time boundary. The article presents the duality between the two time-reversal procedures for waves, and the physical grounds over which each of them stands. While the time-reversal mirrors are based on the application of the Huygens–Fresnel–Helmholtz theorem to predict the field inside a volume, instantaneous time mirrors stem from the Cauchy initial conditions. In this second set-up, time reversal simultaneously acts on the entire space in order to radiate the time-reversed wave, and therefore such a protocol can be considered as a Loschmidt echo for waves.

Overall, the potentiality of echoes to efficiently characterize seemingly disconnected problems, such as quantum phase transitions, non-equilibrium work statistics and parametric correlations of eigenlevels, opens up a very interesting and promising avenue for future research. The present issue, by gathering the state-of-the-art knowledge and understanding of echoes in complex systems, aims to foster such advances.
